# Prediction of effectiveness of universal rotavirus vaccination in Southwestern Vietnam based on a dynamic mathematical model

**DOI:** 10.1038/s41598-024-54775-6

**Published:** 2024-02-21

**Authors:** Taeyong Lee, Ji-Man Kang, Jong Gyun Ahn, Dung Thi Thuy Truong, Thuong Vu Nguyen, Thang Vinh Ho, Ha Thi Thanh Ton, Phuc Le Hoang, Min Young Kim, Joon-Sup Yeom, Jeehyun Lee

**Affiliations:** 1 School of Mathematics and Computing (Mathematics), Yonsei University, 50 Yonsei-ro, Seodaemun-gu, Seoul, 03722 South Korea; 2 Department of Pediatrics, Severance Children’s Hospital, Yonsei University College of Medicine, Seoul, South Korea; 3 Institute for Immunology and Immunological Diseases, Yonsei University College of Medicine, Seoul, South Korea; 4 Department for Disease Control and Prevention, Pasteur Institute, Ho Chi Minh City, Vietnam; 5 Directorial Board, Pasteur Institute, Ho Chi Minh City, Vietnam; 6 Department of Gastroenterology, Children’s Hospital 1, Ho Chi Minh City, Vietnam; 7 Department of Internal Medicine, Severance Hospital, Yonsei University College of Medicine, 50-1 Yonsei-ro, Seodaemun-gu, Seoul, 03722 South Korea

**Keywords:** Rotavirus, Vaccine, Vaccine effectiveness, Dynamic model, Mathematical model, Vietnam, Infectious diseases, Viral infection, Applied mathematics, Gastroenteritis

## Abstract

Vaccinating young children against rotavirus (RV) is a promising preventive strategy against rotavirus gastroenteritis (RVGE). We evaluated the relative risk reduction of RVGE induced by universal vaccination in Vietnam through dynamic model analysis. We developed an age-stratified dynamic Vaccinated-Susceptible-Infectious-Recovered-Susceptible model to analyze RV transmission and assess vaccine effectiveness (VE). We assumed 3 different vaccine efficacies: 55%, 70%, and 85%. For model calibration, we used a database of patients under 5 years of age admitted to Ho Chi Minh No.1 Hospital with RVGE between January 2013 and December 2018. Assuming a vaccination rate of 95%, the number of RVGE hospitalizations after 5 years from universal RV vaccination decreased from 92,502 cases to 45,626 with 85% efficacy, to 54,576 cases with 70% efficacy, and to 63,209 cases with 55% efficacy. Additionally, RVGE hospitalizations after 10 years decreased from 177,950 to 89,517 with 85% efficacy and to 121,832 cases with 55% efficacy. The relative risk reductions of RVGE after 10 years were 49.7% with 85% efficacy, 40.6% with 70% efficacy, and 31.5% with 55% efficacy. The VE was 1.10 times (95% CI, 1.01–1.22) higher in the 4-months to 1-year-old age group than in the other age groups (*P* = 0.038), when applying 85% efficacy with 95% coverage. In conclusion, despite its relatively lower efficacy compared to high-income countries, RV vaccination remains an effective intervention in Southwestern Vietnam. In particular, implementing universal RV vaccination with higher coverage would result in a decrease in RVGE hospitalizations among Vietnamese children under 5 years of age.

## Introduction

Globally, rotavirus (RV) infection remains the main cause of severe acute gastroenteritis in children < 5 years of age, and the majority of fatalities occur in low- and middle-income countries (LMICs)^1–5^. RV vaccination is known to be the best preventive measure for reducing RV-associated morbidity and mortality^6^, but vaccine effectiveness (VE) is affected by several factors such as age, geographical location, seasonality, nutritional status, breastfeeding, co-existing enteropathy, and distribution dynamics of RV genotypes^2,7–10^. Among these, socioeconomic status and sanitation are known to be important factors for the VE of RV vaccines. VE is as high as 85–98% in high-income countries and as low as 51–64% in LMICs^8,11,12^. The relatively low effectiveness in LMICs is one reason for policymakers’ reluctance to aggressively introduce RV vaccines in their national immunization program (NIP)^13–15^. However, due to the high disease burden and evident VE, the World Health Organization strongly recommends including the RV vaccine in NIPs and gives priority to some geographic regions, especially Southern and Southeast Asia^16,17^, and Sub-Saharan Africa^18^.

Vietnam is one of the countries with a high disease burden of RV infection, but the RV vaccine has not been included in Vietnam's NIP up to October 2022, despite the presence of initial comprehensive multi-year plans. We recently reported the epidemiology of rotavirus gastroenteritis (RVGE) and RV VE in Southern Vietnam between 2013 and 2018^5^. In this hospitalization-based surveillance data, we found that RV vaccine coverage was low (4%), and the overall VE was as low as 59–70%. In addition, the RVGE incidence rate was lower in the Southeastern region, characterized by a higher socioeconomic status including a higher gross domestic product and lower child mortality rate, than in the Southwestern region, characterized by a lower socioeconomic status. However, mathematical studies that are based on the latest epidemiological research and evaluate the impact of RV vaccine introduction in the NIP of Vietnam are lacking^19^. In this study, we analyzed the impact of VE on the reduction of RVGE hospitalization in Vietnam at the national level using a dynamic mathematical model.

## Methods

We designed a dynamic compartmental model of RVGE infection and transmission in Vietnam. Age-specific characteristics and demographic changes in the Vietnamese population were incorporated using a structured model. The parameter values in the model were determined based on a literature review or calibrated using epidemiologically relevant data. We then estimated the hospitalization rate due to RVGE under different vaccination scenarios by varying the vaccine efficacy and coverage.

An exemption for ethical approval was obtained from the Institutional Review Board of Yonsei University (4-2021-0546). All methods were performed in accordance with the relevant guidelines and regulations including, but not limited to, those set by the Institutional Review Board of Yonsei University.

### Mathematical model and population structure

A dynamic compartmental model divides the total population into several groups according to disease status. There have been several studies to investigate the RV spreads with this compartmental model. Using a model allowing multiple infection, Pitzer et al.^20^ showed that birth rate differences in United States have a strong influence on RV epidemics and predicted a pattern of epidemics after vaccine introduction. Applying and transforming this model, there have been various research analyzing the rotavirus dynamics in various regions, such as, New York City^21^, Belgium^22^, Malawi^23^, and Ghana^24^.

**Figure 1:**
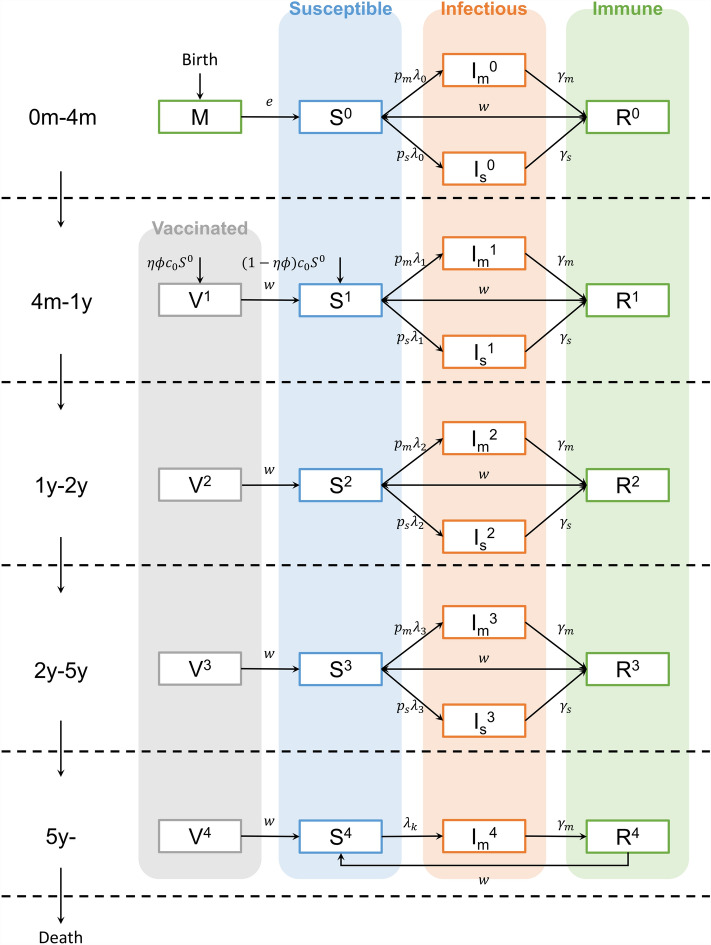
Diagram of age-stratified Vaccinated-Susceptible-Infectious-Recovered-Susceptible model. The first age group labeled as “0m-4m” represents individuals aged equal to or above 0 months but below 4 months, and the same principle applies to the other groups. The final group signifies individuals aged 5 years and above. The demographic change and disease status are described by the flows. The population aged over five years depleted through the death rate, assuming an annual death rate equivalent to the birth rate. By employing demographic data of the Vietnamese population, annual population recalibration was conducted to reflect population changes over time. All populations are aging with the rate of $c$, which is determined by the length of age group. Infants less than 4 months of age have maternal immunity from birth (M); however, they will become susceptible with rate, $e$, which is the reduction of transplacental protective immunity. The susceptible groups (S) are infected RV with the force of infection, $\lambda$, and will have mild symptoms with the probability, $p_{m}$, and severe symptoms with the probability, $p_{s}$. Each of them is recovered with the rate of recovery rate, $\gamma_{m}$ and $\gamma_{s}$, respectively. After recovery, they can be re-infected because of waning of immunity. The waning rate is described as the parameter, $w$. The infants less than 4 months are vaccinated when they become old with the efficacy $\eta$ and coverage $\phi$. The subindices stand for the age-specific parameters. This transmission dynamic occurs for all ages with age-specific parameters.

Based on the models of previous studies, we designed a dynamic compartmental model to describe the rotavirus dynamics reflecting demographic changes in Vietnam. By dividing the population into 6 different disease status; maternal immunity, vaccinated, susceptible, infectious with mild symptoms, infectious with severe symptoms, and recovered, we described the RV infection status. Moreover, by stratifying the population 5 age groups, the model explained population structure changes. The detailed flows for each compartment are drawn in the Fig. 1 and Table 1^25^. It allows reinfections due to waning immunity of recovered and vaccinated people and maternal immunity protecting infants younger than 4 months^26^.

**Table 1:** Summary of model parameters.

Parameters	Symbol	Value	Source
Waning rate of maternal immunity	$e$	$1/0.5$ years^−1^	Assumed
Recovery rate of mildly symptomatic case	$\gamma_{m}$	$1/4.5$ days^−1^	^30^
Recovery rate of severely symptomatic case	$\gamma_{s}$	1/8 days^−1^	^30^
Proportion of mildly symptomatic case	$p_{m}$	$$\left\{ {\begin{array}{*{20}c} {\frac{109}{{111}} ( < 5y.o.)} \\ {1 \left( { \ge 5y.o.} \right)} \\ \end{array} } \right.$$	^31^
Proportion of severely symptomatic case	$p_{s}$	$1 - p_{m}$	^31^
Waning rate of immunity	$w$	${\text{ln}}2/2{ }$ years^−1^	^32^
Who-Acquires-Infection-From-Whom matrix	$W$		Estimated
	$\beta_{1}$	$9.95 \times 10^{ - 7} \left[ {7.21 \times 10^{ - 7} ,\;\; 1.22 \times 10^{ - 6} } \right]$	
	$\beta_{2}$	$6.86 \times 10^{ - 7} \left[ {6.01 \times 10^{ - 7} ,\;\;7.83 \times 10^{ - 7} } \right]$	
	$\beta_{3}$	$5.29 \times 10^{ - 19} { }\left[ {0,{ }\;\;3.00 \times 10^{ - 7} } \right]$	
	$\beta_{4}$	$1.25 \times 10^{ - 8} { }\left[ {0,{ }\;\;2.10 \times 10^{ - 8} } \right]$	
	$\beta_{5}$	$1.47 \times 10^{ - 21} { }\left[ {0,{ }\;\;5.91 \times 10^{ - 8} } \right]$	
	$\beta_{6}$	$1.28 \times 10^{ - 8} { }\left[ {4.68 \times 10^{ - 9} , \;\;2.80 \times 10^{ - 8} } \right]$	
Amplitude of force of infection	$A$	$- \;9.62 \times 10^{ - 3} { }\left[ { - \;1.64 \times 10^{ - 2} ,{ }\;\; - \;6.61 \times 10^{ - 3} } \right]$	Estimated
Offset of force of infection	$\theta$	$- \;41.1{ }\left[ { - \;87.4,{ }13.8} \right]$	Estimated

If the parameter is estimated, profile likelihood 95% confidence interval is also presented.

In modeling, complete vaccination was defined as a 2-dose schedule of Rotarix or Rotavin M-1, the locally licensed, G1P[8] strain-based RV vaccine with a similar protective effect^8^. Therefore, we assumed that infants are vaccinated with the RV vaccine at around 4 months of age, and only susceptible individuals are affected. The immunity of individuals who have been vaccinated or have recovered gradually wanes. Those who have lost immunity among the vaccinated and recovered individuals, as well as those who are not infected, are susceptible and can become infected. Infected individuals develop mild or severe symptoms. We assumed that infected patients over 5 years of age are not hospitalized with RVGE because their symptoms are mild^27,28^.

To incorporate age-specific characteristics of RV that severely affect young children under the age of 5 years, the model was stratified into 5 age groups, as shown in Fig. 1. The change in the age structure of the population was also considered, which plays an important role in RV transmission. We referred to the Vietnamese population data from 2010 to 2040. Data from 2010–2019 was obtained from the General Statistics Office of Vietnam^29,30^, and we used projected population data after 2019 from the United Nations data^31^. Observing the population structure through time, we found a sharp decline in the proportion of the population aged 0–5 years compared to other age groups (Fig. S1). When we integrate all population information, the proportion of children aged < 5 years was approximately 8% of the total population from 2010 to the early 2020s; however, it is predicted to decrease by approximately 6% in the 2030s. These populations were reflected in the model with adjusting the model population in simulation.

The infectivity among the population should be estimated. Using epidemiological data, it is a standard approach to formulate a Who-Acquires-Infection-From-Whom (WAIFW) matrix with a mathematical model^32^, which describes transmission in a heterogeneous population, whose structure is represented in Eq. (1). Taking into account the characteristics of rotavirus transmission, we chose to impose structure on the WAIFW matrix instead of assuming direct proportionality to the contact rate. WAIFW is constructed symmetrically with transmission rates, $\beta_{1} , \beta_{2} , \ldots ,\beta_{6} ;$ its component, denoted by $W_{ij}$, means the transmissibility of infectious people belonging to the $j$-th age group to susceptible people belonging to the $i$-th age group. To reflect the seasonality of infection, a trigonometric function was employed in the WAIFW matrix^32^.

1 $$W = \left( {1 + A{\text{cos}}\left( {\frac{2\pi }{{365}}\left( {t - \theta } \right)} \right)} \right)\left[ {\begin{array}{*{20}c} {\beta_{1} } & {\beta_{1} } & {\beta_{2} } & {\beta_{5} } & {\beta_{6} } \\ {\beta_{1} } & {\beta_{1} } & {\beta_{2} } & {\beta_{5} } & {\beta_{6} } \\ {\beta_{2} } & {\beta_{2} } & {\beta_{2} } & {\beta_{5} } & {\beta_{6} } \\ {\beta_{5} } & {\beta_{5} } & {\beta_{5} } & {\beta_{3} } & {\beta_{6} } \\ {\beta_{6} } & {\beta_{6} } & {\beta_{6} } & {\beta_{6} } & {\beta_{4} } \\ \end{array} } \right]$$

is the amplitude of oscillation of infectivity, and $\theta$ is the offset.

The force of infection at time $t$, $\lambda \left( t \right)$, can be written as:

2 $$\lambda \left( t \right) = W\left( {qI_{m} \left( t \right) + I_{s} \left( t \right)} \right)$$

$q$ represents the reduced risk of infection for patients with mild symptoms ($I_{m}$) relative to those with severe symptoms ($I_{s}$), whose value is assumed to be 0.5. Susceptible people who become infected develop mild symptoms with a probability $p_{m}$, and severe symptoms with a probability $p_{s}$. It is assumed that only severe cases are hospitalized, which leads $p_{m}$ to equal 1 for susceptible people over 5 years old because they are usually not hospitalized with RVGE. We described additional details in the text S1.

### Model calibration and data source

The parameter values in the model were determined based on a literature review or calibrated to relevant information. The descriptions, values, and references for the parameters are summarized in Table 1^36–38^. From the literature review, we could determine various epidemic parameters about disease status. However, parameters related to infection, such as $\beta_{1} ,\beta_{2} , \ldots ,\beta_{6} ,A,\theta$, should be estimated with hospitalization data in Vietnam. Waning period of maternal immunity was assumed to be 6 months on average. We set the hospitalization proportion by combining the results of the Ho Chi Minh City and Dong Thap regions investigated in a previous study^39^. For estimation, we used an anonymized database of patients aged < 5 years admitted with acute gastroenteritis to Pediatric Hospital No.1 in Ho Chi Minh City, Vietnam, from January 2013 to December 2018^5^.

Maximum likelihood estimation was applied assuming Poisson-distributed data and the following likelihood function (Fig. S2):

3 $${\mathcal{L}} = \Pi_{i, j} {\text{Prob}}\left\{ {\xi_{i, j} = \xi_{i, j}^{*} {|}\xi_{i,j} \sim {\text{Poisson}}\left( {\zeta_{i, j} } \right)} \right\}$$

In this formula (3), $\xi_{i,j}^{*}$ is the monthly hospitalization data for $i$-th age group at $j$-th month from January 2013 to December 2019. $\zeta_{i,j}$ is the expected number of monthly hospitalized people in the model calculated by $\zeta_{i, j} = h_{k\left( j \right)} \mathop \smallint \limits_{j} p_{s} \lambda_{i} \left( t \right)S_{i} \left( t \right)dt$ with hospitalization rate $h_{k\left( j \right)}$. The profile likelihood-based confidence region of the parameters was obtained to display the effect of uncertainty on the incidence. The results of parameter estimation are presented in Table 1.

### Vaccination scenarios and sensitivity analysis

We set 95% coverage of the RV vaccine in Vietnam as the main scenario and summarize the effects of RV vaccine with the 65%, 75%, and 85% coverage. To investigate the effect of vaccine efficacy on hospitalized patients, we assumed three different efficacies: 55%, 70%, and 85%. In the model, the effective vaccination proportion is implemented as the product of coverage and efficacy. All scenarios were simulated from 2019 to estimate the transmission of RV, assuming that vaccination begins at January 1, 2023. It meant the initial incorporation of the RV vaccine into the NIP on the first day of 2023. Therefore, we analyzed the cumulative numbers of hospitalizations from that date. To examine the impact of VE on RV transmission, we estimated the age-specific incidence under 3 scenarios of varying efficacy assuming coverage as 65%, 75%, 85%, and 95%.

Predicting the impact of uncertainty in parameter estimation on the number of inpatients by age is not straightforward. We used 95% confidence intervals of the estimated parameters to observe uncertainty in hospitalization dynamics. By sampling 1,000 parameter sets from the confidence intervals and simulating our dynamic model for each set, we predicted the future dynamics of hospitalization for each age group with a possible range. To observe VE, we calculated relative risk reductions for 5 years and 10 years after the initial introduction of the vaccination policy. Moreover, using the predictive profile likelihood method, these values are presented with uncertainties.

### Compliance with ethics guidelines

We obtained an exemption from the Institutional Review Board of Yonsei University (4-2021-0546). The need for informed consent was waived by the Institutional Review Board of Yonsei University owing to the retrospective nature of the study.

## Results

We predicted the number of hospitalization cases for RVGE in each age group from January 2019 to December 2032 with no-vaccination scenario and its dynamics can be seen in Fig. S3. The uncertainty ranges are also presented. Without RV vaccination in the NIP, RVGE hospitalization was predicted to range from 74,125 to 118,270 with an average of 92,502 for 5 years, and from 138,670 to 230,480 with an average of 177,950 for 10 years. Under the no-vaccination scenario, the incidence remained at a similar level, and uncertainty increased.

### Vaccine effectiveness

**Figure 2:**
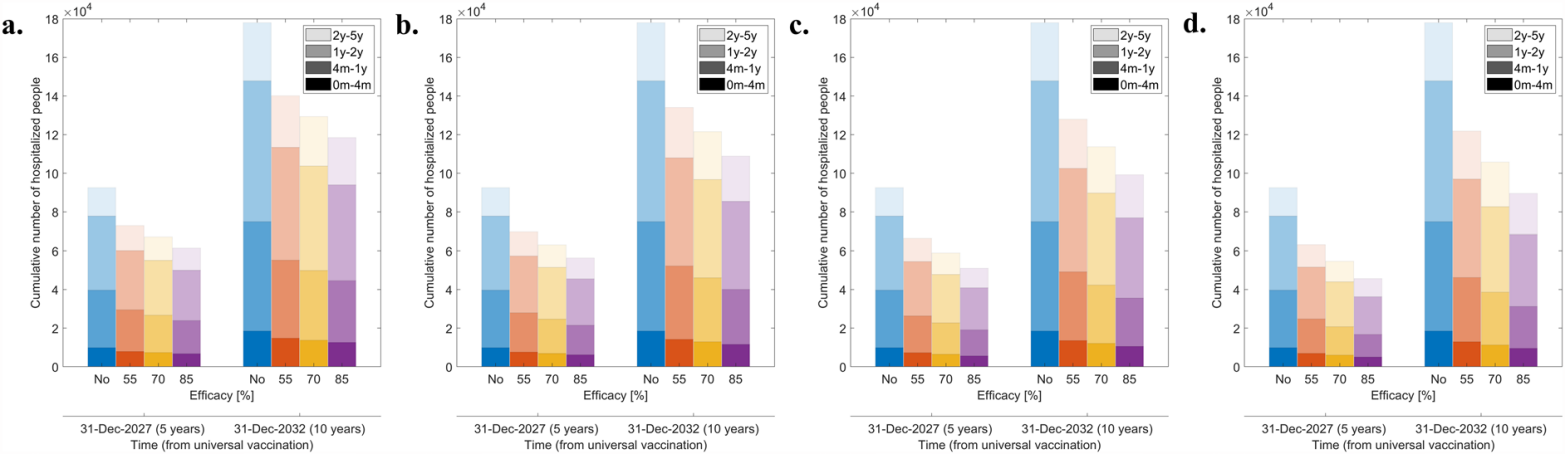
Cumulative number of hospitalized patients by age with different vaccine efficacies for 5 years versus 10 years post-vaccination. Vaccine coverage is (**a**) 65%, (**b**) 75%, (**c**) 85%, and (**d**) 95%. Each color of bar represents vaccine efficacy assumption; blue: no vaccination, red: 55%, yellow: 70%, and purple: 85%. The first age group labeled as “0m-4m” represents individuals aged equal to or above 0 months but below 4 months, and the same principle applies to the other groups. The final group signifies individuals aged 5 years and above.

We compared cumulative RVGE hospitalizations over the first 5 and 10 years for various vaccine efficacies and no-vaccination scenarios (Fig. 2a–d). Results according to efficacy exhibited similar trends across various vaccine coverage scenarios. Among these, the specific figures for a 95% coverage rate (summarized in Table 2) reveal a significant impact on children under the age of 2. For a vaccine efficacy of 55%, a reduction of approximately 30% was predicted over a 10-year period for the youngest age group and for patients aged between 1 and 2 years. When efficacy increased to 70%, the risk reduction rate increased to 40%, and at 85% efficacy, it rose to 49.0%. These results were similar in other groups, with the vaccine being most effective in those aged between 4 months and 1 year.

**Table 2:** The relative risk reduction of the rotavirus gastroenteritis hospitalization in children less than 5 years old after the introduction of rotavirus vaccine into the national immunization program in Vietnam compared with the scenario without vaccine introduction.

Ages of population	Vaccination period	Vaccine efficacy case^a b^		
		55%	70%	85%
All (0–5 years)	5 years	31.7% (31.2–32.7%)	41.0% (40.6–42.3%)	50.7% (49.7–50.8%)
	10 years	31.5% (31.5–32.5%)	40.6% (40.4–40.6%)	49.7% (49.7–49.8%)
0–4 months	5 years	29.8% (29.1–30.9%)	39.0% (37.7–40.7%)	48.9% (48.1–52.4%)
	10 years	30.1% (29.4–32.3%)	39.2% (38.3–42.3%)	48.5% (47.9–52.4%)
4–12 months	5 years	40.2% (39.8–41.4%)	50.7% (50.1–51.2%)	60.9% (60.6–63.6%)
	10 years	41.1% (40.5–41.9%)	51.6% (50.8–53.4%)	61.5% (61.5–63.4%)
1–2 years	5 years	29.8% (29.1–31.6%)	39.0% (38.2–40.2%)	48.9% (48.2–52.0%)
	10 years	30.3% (29.7–30.7%)	39.5% (38.3–39.5%)	49.0% (47.5–52.9%)
2–5 years	5 years	20.7% (20.2–23.0%)	27.9% (27.0–30.7%)	35.9% (34.8–37.8%)
	10 years	17.5% (16.9–19.9%)	23.4% (23.4–24.4%)	30.0% (29.6–32.4%)

^a^The numbers in parentheses are the 95% prediction confidence intervals.

^b^Here—we assume that the vaccination coverage is 95%.

The results for various vaccine coverage scenarios (Fig. 2) show that although the overall trends in the efficacy-specific graphs appear similar with varying vaccine coverage, a quantitative analysis reveals differences. When a vaccine with 55% efficacy is administered with a 65% coverage rate, the number of patients aged below 4 months reduced approximately 20% over a 10-year period (Fig. 2a). With vaccine efficacies of 70% and 85%, the reduction in patients in this age group is 26% and 36%, respectively. A coverage rate of 75% results in reductions corresponding to the efficacy of each vaccine, with values of 23%, 30%, and 37% (Fig. 2b). Assuming an 85% coverage rate, the incidence in the youngest group decreases by 26%, 35%, and 43% for each vaccine efficacy (Fig. 2c). Across all age groups, as coverage rates increase, the burden of the disease decreases.

**Figure 3:**
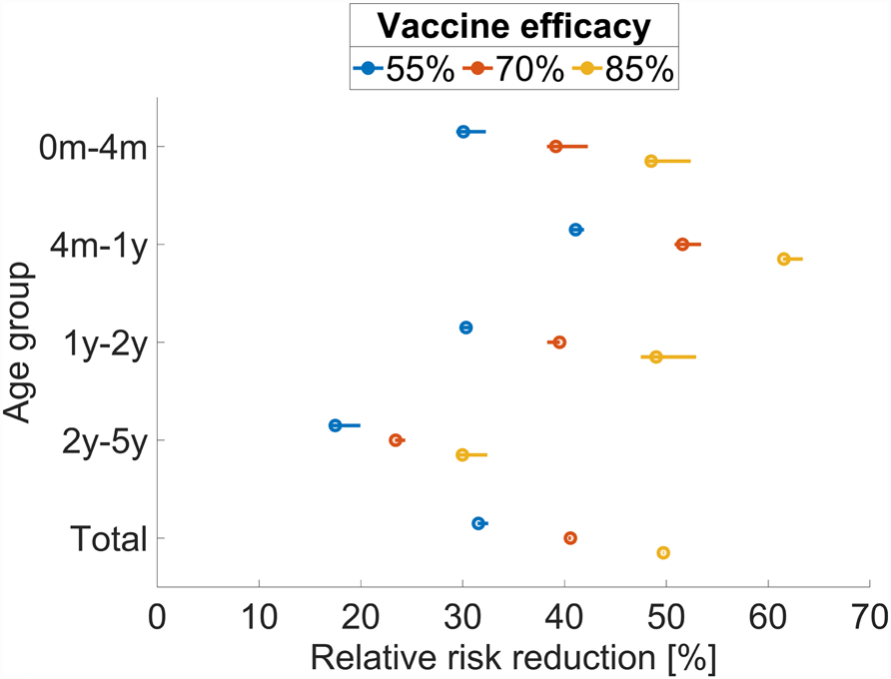
Relative risk reductions in the hospitalization rates under different vaccine efficacies compared to the baseline of no vaccination after 10 years from the introduction of the vaccine. The line for each point means the 95% predictive confidence interval of relative risk reduction. The first age group labeled as “0m-4m” represents individuals aged equal to or above 0 months but below 4 months, and the same principle applies to the other groups. The final group signifies individuals aged 5 years and above.

Assuming a vaccination coverage rate of up to 95%, a 10-year vaccination program with maximum efficacy reduced RVGE hospitalizations by 49.7% (95% CI, 49.7–49.8%) and one with minimum efficacy reduced RVGE hospitalizations by 31.5% (95% CI, 31.4–32.5%; Table 2). Vaccines are especially beneficial for children aged 4 months to 1 year, which is the most vulnerable age group. For example, the relative risk reduction of a 10-year vaccination policy with 85% efficacy and 95% coverage for this age group is 1.10 (95% CI 1.01–1.22) times higher (p-value 0.0384) than the one for the other children (Figs. 3 and S4).

We used the merged diarrheal hospitalization rate of the two different regions: Dong Thap Province and Ho Chi Minh City. When we applied it separately, we had several different results in the RVGE incidence rate and transmission dynamics. Using a diarrheal hospitalization rate in Dong Thap Province (126/2199.4 cases per IYO (infants-year of observation)) whose socioeconomic status and sanitation level is relatively lower than Ho Chi Minh City case, 4.31 cases per 10,000 people less than 5 years old are predicted to be infected for a month. However, with the Ho Chi Minh City’s hospitalization rate (18/4040 cases per IYO), we had 0.43 cases, which was about 10 folds lower (p-value < 0.001). Moreover, the transmission pattern was also different, and you can see the details at the Fig. S5.

## Discussion

We predicted that universal RV vaccination in Vietnam reduces RVGE hospitalizations in children under 5 years of age using a dynamic mathematical model. Although VE against RVGE hospitalization varies with 30–50 in our study, children aged 4 months to 2 years had the highest VE. In particular, it was predicted that even infants under 4 months of age, the most vulnerable age group, could receive a protective effect even though they could not complete the RV vaccination schedule.

Worldwide, it is estimated that approximately 1.5 million hospitalizations and 130,000 deaths occur due to RVGE in children under 5 years of age, and most of the occur in LMICs that do not include the RV vaccine in their NIPs^6,33^. Moreover, a 26–40% reduction in RVGE in children under 5 years of age was reported in LMICs that introduced RV vaccination to the NIP through prospective RVGE hospitalization real-world surveillance data. The age of RVGE-hospitalized children increased after introduction of the RV vaccine^34^.

However, in their meta-regression study, Clark et al.^35^ reported that the effectiveness of the RV vaccine for the prevention of severe RVGE in LMICs was only 44% (95% CI, 27–59%) after 12 months compared to 94% (95% CI, 87–98%) in high-income countries. However, the effect of RV vaccination tended to remain relatively high at 61–81% until the first 3 months after completion of vaccination in all countries excluding India. Nevertheless, RV vaccines are an effective and important preventive measure even in LMICs. Therefore, a protective effect on young infants who are more vulnerable to severe RVGE can be expected. Thus, the relatively weak effect of RV vaccination predicted in this study, compared to the efficacy in high-income countries, is not an appropriate reason for the reluctance to include RV in the NIP in Vietnam. Kraay et al.^40^ reported that rotavirus vaccination may be less effective in low- and middle-income countries (LMICs) than in high-income countries due to indirect effects, but it was effective against severe diseases in all 112 countries evaluated, regardless of income level. Additionally, we already reported that the RVGE hospitalization rate was lower in the southeastern region of Vietnam, which has a higher socioeconomic status, including a higher GDP and a lower child mortality rate, than in the southwestern region. These findings emphasize the importance of improving both public health and sanitation, as well as socioeconomic status, to reduce the burden of RVGE^5^.

Vietnam, in particular, is a country that is developing rapidly, and RV vaccine introduction is predicted to have a greater value in the future than that predicted in this study. We made more accurate predictions by reflecting the age structure of the Vietnamese population using a dynamic model. The majority of existing RV VE prediction studies or cost-effectiveness studies have used static models. However, since this does not reflect the increase or decrease in the susceptible population according to the change in the population structure, results can be overestimated or underestimated. According to a study published by Russell et al.^41^ on the efficacy of maternal vaccination for neonatal pertussis, the static model reported that maternal vaccination was cost-effective in all cases. However, the dynamic model, reported that if the vaccination rate for infants and children is high and herd immunity is high, maternal vaccination would not be cost-effective. In this study, we applied the demographic prediction data of United Nations, in which the fraction of the population under the age of five, which is the susceptible population for RV, is gradually decreasing. Therefore, our results are less likely to be overestimated compared to the static model^19^.

When selecting the type of vaccine to be introduced into the NIP, the efficacy of the vaccine against current or future epidemic strains in the country is essential. Therefore, we tried to reflect the latest RVGE epidemic as much as possible by performing sensitivity analysis to the data of patients hospitalized for RVGE in Southern Vietnam collected in 2013–2018. G1P[8] based vaccines that are likely to be introduced in Vietnam had direct or cross-protective effects on G3P[8], G8P[8], and G1P[8], which were the most prevalent strains in Vietnam during the 2013–2018 study period^5,42^. Meanwhile, in the case of the G2 strain, including G2P[4], the protective effect against RV is lower than in other prevalent strains^8^. Particularly, there are reports showing that G2P is emerging as one of the major strains after the massive RV vaccination^43,44^. Therefore, in a previous study in Vietnam, the number of cases with G2P strains was as low as four; however, continuous RV genotype monitoring is needed^5^.

RV transmission occurs via the fecal–oral route and is closely related to hygiene. We developed an age-structured model to incorporate age-specific characteristics of the RV transmission, in which the transmission matrix represented transmission rates between different age groups. The structure of the transmission matrix was assumed to describe RV transmission dynamics in Vietnam. The seasonality of RV infection dynamics was explained by using a trigonometric formula. Our model reflected the changing demographics of Vietnam because the impact of vaccination could not be properly estimated without considering this change^31^.

Our study has some limitations. First, the single-center data in Ho Chi Minh City, Vietnam, used to calibrate the results of this study were collected from the Southwestern region, and there is a limit to representing Vietnam as a whole. Second, due to the limitations of the existing literature and data, it was possible to measure only the hospitalization effect of RVGE rather than the prevention of RV infection itself. Third, for certain parameters for which there is a lack of local data from Vietnam, we cited existing literature and data from other LMICs, which may not accurately represent the true characteristics of RVGE in Vietnamese children. Finally, a cost-effectiveness analysis could not be conducted. However, our study has strengths, as it is the first to analyze the effects of RVGE in Vietnam through a dynamic model. In addition, we tried to reflect the local situation in Vietnam as much as possible based on data collected prospectively from children under the age of 5 years who were hospitalized, with more than 5,000 acute gastroenteritis cases.

## Conclusions

Universal RV vaccination, which can induce high vaccination coverage, can significantly reduce hospitalizations for RVGE in children under 5 years of age in Vietnam. With analyses using an age-stratified dynamic model, we could predict that, with RV vaccine, the effects would be the best for the most vulnerable children aged of 4 months–1 year. In addition, modest protection can also be expected for infants younger than 4 months of age who cannot be fully vaccinated with the RV vaccine. Certainly, our results are largely speculative based on several assumptions; however, this study has the aspect of being able to estimate the effect of the universal RV vaccination, which requires a considerable budget and medical resources in advance. As a universal RV vaccine is expected to be introduced in Vietnam shortly, we expect that it will be possible to evaluate how similar or different the actual effect of RV vaccination is in the near future. In addition, the mathematical model and predictions in this study could serve as a reference for the implementation of universal RV vaccination in other LMICs where such vaccination is not currently underway.

### Supplementary Information

Supplementary Information.

## Data Availability

All data generated or analyzed during this study are included in the published article^5^. The datasets used and/or analyzed during the current study are available from the corresponding author on reasonable request.

## Abbreviations

RV: Rotavirus
RVGE: Rotavirus gastroenteritis
VE: Vaccine effectiveness
LMIC: Low- and Middle-income countries
NIP: National immunization program
WAIFW: Who-acquires-infection-from-whom
IYO: Infants-year of observation
