# Supplementary material for: Prediction of effectiveness of universal rotavirus vaccination in Southwestern Vietnam based on a dynamic mathematical model
Source: Sci Rep. 2024 Feb 21;14:4273. doi: 10.1038/s41598-024-54775-6 (PMC10881495; doi:10.1038/s41598-024-54775-6)
Supplement: Supplementary file 1 — Supplementary Information. [file 41598_2024_54775_MOESM1_ESM.pdf]

Prediction of effectiveness of universal rotavirus vaccination in Southwestern Vietnam based on a dynamic mathematical model

Taeyong Lee[a*], Ji-Man Kang[b,c*], Jong Gyun Ahn[b,c], Dung Thi Thuy Truong[d], Thuong Vu Nguyen[e], Thang Vinh Ho[d], Ha Thi Thanh Ton[f], Phuc Le Hoang[f], Min Young Kim[b,c], Joon-Sup Yeom[g**], Jeehyun Lee[a**]

[a]School of Mathematics and Computing (Mathematics), Yonsei University, Seoul, South Korea

[b]Department of Pediatrics, Severance Children's Hospital, Yonsei University College of Medicine, Seoul, South Korea

[c]Institute for Immunology and Immunological Diseases, Yonsei University College of Medicine, Seoul, South Korea

[d]Department for Disease Control and Prevention, Pasteur Institute, Ho Chi Minh City, Vietnam

[e]Directorial Board, Pasteur Institute, Ho Chi Minh City, Vietnam

[f]Department of Gastroenterology, Children's Hospital 1, Ho Chi Minh City, Vietnam

[g]Department of Internal Medicine, Severance Hospital, Yonsei University College of Medicine, Seoul, South Korea

[*]Co-first authors: Taeyong Lee and Ji-Man Kang contributed equally to this work.

[**]Co-corresponding authors: Joon-Sup Yeom and Jeehyun Lee contributed equally to this work.

<u>**Address for Correspondence:**</u>

Professor Joon-Sup Yeom, MD, DTM&H, PhD

50-1 Yonsei-ro, Seodaemun-gu, Seoul 03722, South Korea

Tel: 82-2-2228-1942

E-mail: JOONSUP.YEOM@yuhs.ac

Prof Jeehyun Lee, Ph.D

50 Yonsei-ro, Seodaemun-gu, Seoul 03722, South Korea

Telephone: 82-2-2123-5581

E-mail: ezhyun@yonsei.ac.kr

Appendix

Figure S1. Population dynamics (left axis) for young children under 5 years old and (right axis) for the people equal to or 5 years old.

Figure S2. Parameter estimation results: the dots represent the number of monthly hospitalizations per 10,000 capita and the line plots the expected number of hospitalized people predicted by the model with a shaded region owing to the 95% confidence interval. The first age group labeled as "0m-4m" represents individuals aged equal to or above 0 months but below 4 months, and the same principle applies to the other groups. The final group signifies individuals aged 5 years and above.

Figure S3. Prediction of the age-specific incidence rates of rotavirus gastroenteritis hospitalizations from 2019 to 2032. The first age group labeled as "0m-4m" represents individuals aged equal to or above 0 months but below 4 months, and the same principle applies to the other groups. The final group signifies individuals aged 5 years and above. The solid lines show the average incidence rate for each age group with the estimated parameters. The shades, on the other hand, depict the range between minimum and maximum incidence from the profile-likelihood-based confidence ranges of parameters.

Figure S4. Age-specific RVGE incidence rate under 5 years old. The first age group labeled as "0m-4m" represents individuals aged equal to or above 0 months but below 4 months, and the same principle applies to the other groups. The final group signifies individuals aged 5 years and above.

Figure S5. Prediction of the age-specific incidence rates of rotavirus gastroenteritis hospitalizations from 2019 to 2032 with the hospitalization rate from (A) Ho Chi Minh City and (B) Dong Thap Province. The first age group labeled as "0m-4m" represents individuals aged equal to or above 0 months but below 4 months, and the same principle applies to the other groups. The final group signifies individuals aged 5 years and above. The dashed lines show the average incidence rate for each age group with the estimated parameters. The shades, on the other hand, depict the range between minimum and maximum incidence from the profile-likelihood-based confidence ranges of parameters.

Text S1. Mathematical model details to describe Vietnamese rotavirus dynamics.

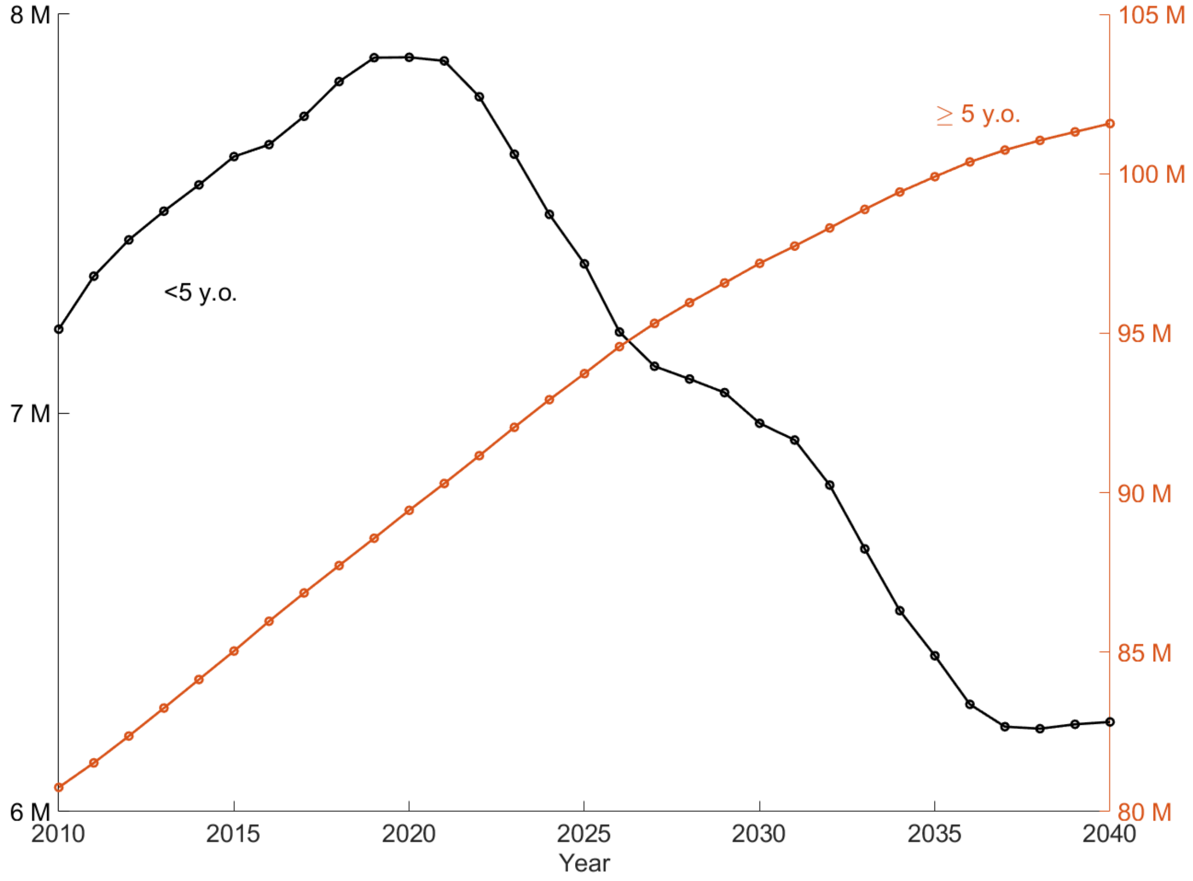

Figure S1. Population dynamics (left axis) for young children under 5 years old and (right axis) for the people equal to or 5 years old.

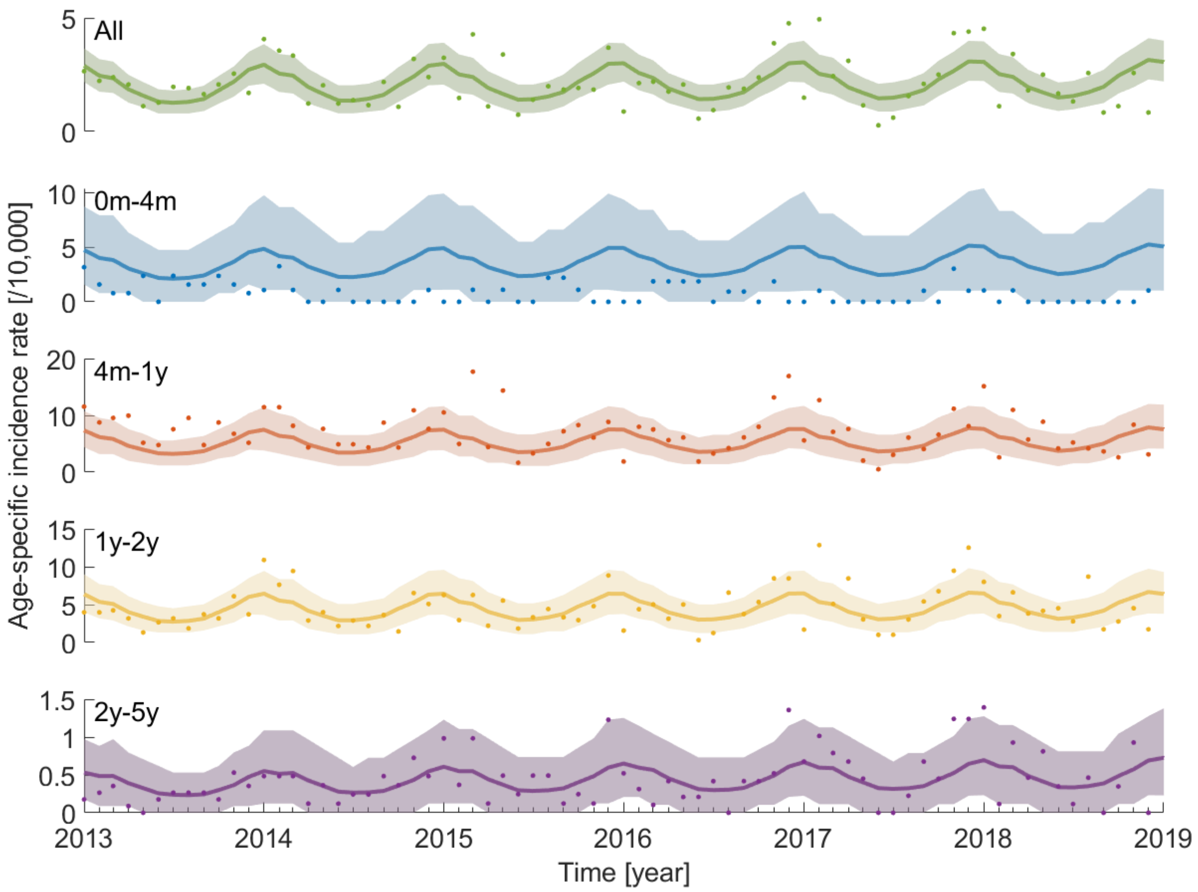

Figure S2. Parameter estimation results: the dots represent the number of monthly hospitalizations per 10,000 capita and the line plots the expected number of hospitalized people predicted by the model with a shaded region owing to the 95% confidence interval. The first age group labeled as "0m-4m" represents individuals aged equal to or above 0 months but below 4 months, and the same principle applies to the other groups. The final group signifies individuals aged 5 years and above.

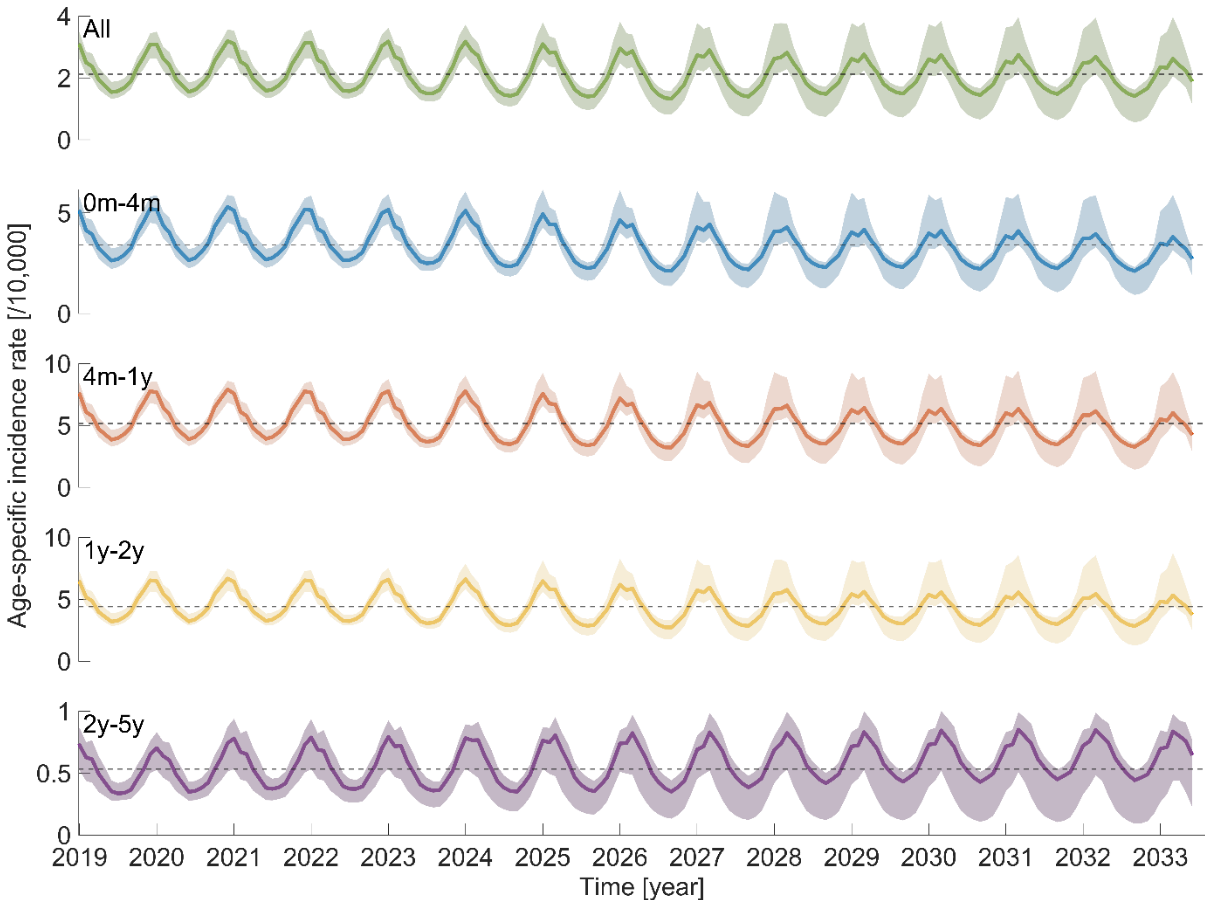

Figure S3. Prediction of the age-specific incidence rates of rotavirus gastroenteritis hospitalizations from 2019 to 2032. The first age group labeled as "0m-4m" represents individuals aged equal to or above 0 months but below 4 months, and the same principle applies to the other groups. The final group signifies individuals aged 5 years and above. The dashed lines show the average incidence rate for each age group with the estimated parameters. The shades, on the other hand, depict the range between minimum and maximum incidence from the profile-likelihood-based confidence ranges of parameters.

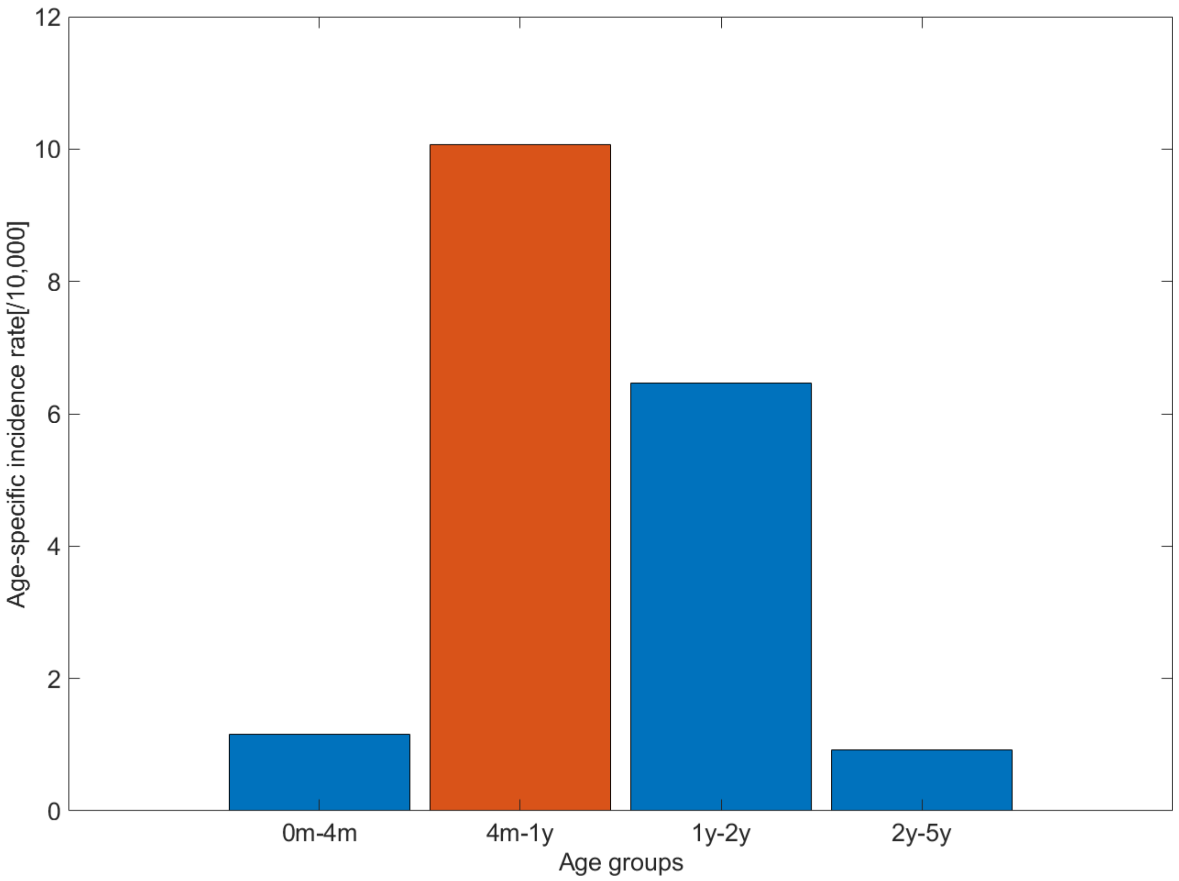

Figure S4. Age-specific RVGE incidence rate under 5 years old. The first age group labeled as "0m-4m" represents individuals aged equal to or above 0 months but below 4 months, and the same principle applies to the other groups. The final group signifies individuals aged 5 years and above.

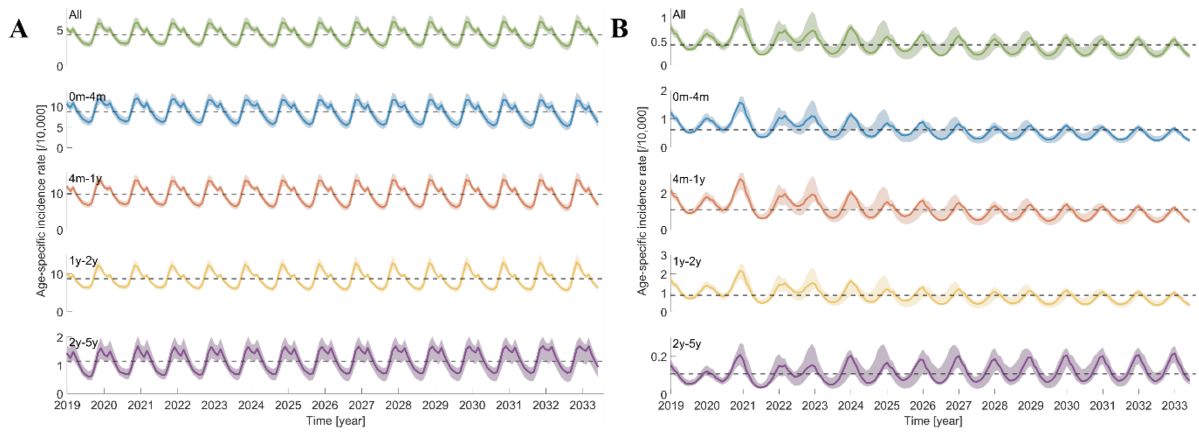

Figure S5. Prediction of the age-specific incidence rates of rotavirus gastroenteritis hospitalizations from 2019 to 2032 with the hospitalization rate from (A) Ho Chi Minh City and (B) Dong Thap Province. The first age group labeled as "0m-4m" represents individuals aged equal to or above 0 months but below 4 months, and the same principle applies to the other groups. The final group signifies individuals aged 5 years and above. The dashed lines show the average incidence rate for each age group with the estimated parameters. The shades, on the other hand, depict the range between minimum and maximum incidence from the profile-likelihood-based confidence ranges of parameters.

Text S1. Mathematical model details to describe Vietnamese rotavirus dynamics.

We constructed a differential equations system to describe the dynamics of rotavirus in response to vaccines in Vietnam. The detailed differential equations are shown below. Given that some ages are 0 years old, the indices for the force of infection λ and the aging c start from 0 and are represented as subindex. For each year, the number of births is B_{year} and the total number of deaths is the same. First, we wrote the dynamics for the youngest people aged from 0 to 4 months as follows.

$$\begin{cases} \dfrac{dM}{dt} = B_{\text{year}} - eM - c_0 M \\ \dfrac{dS^0}{dt} = eM - (p_m + p_s)\lambda_0 S^0 + wR^0 - c_0 S^0 \\ \dfrac{dI_m^0}{dt} = p_m \lambda_0 S^0 - \gamma_m I_m^0 - c_0 I_m^0 \\ \dfrac{dI_s^0}{dt} = p_s \lambda_0 S^0 - \gamma_s I_s^0 - c_0 I_s^0 \\ \dfrac{dR^0}{dt} = \gamma_m I_m^0 + \gamma_s I_s^0 - wR^0 - c_0 R^0 \end{cases}$$

The people aged from 4 months to a year are vaccinated when they are aged from the previous compartment. Therefore, the aging processes for the vaccinated and susceptible groups are slightly different than the others.

$$\begin{cases} \dfrac{dV^1}{dt} = -wV^1 + \eta\phi c_0 S^0 + c_0 M - c_1 V^1 \\ \dfrac{dS^1}{dt} = -(p_m + p_s)\lambda_1 S^1 + w(V^1 + R^1) + (1 - \eta\phi) c_0 S^0 - c_1 S^1 \end{cases}$$

For the older age classes, the vaccinated and susceptible people have the following dynamics instead.

$$\begin{cases} \dfrac{dV^i}{dt} = -wV^i + c_{i-1} V^{i-1} - c_i V^i \ (i = 2, 3) \\ \dfrac{dS^i}{dt} = -(p_m + p_s)\lambda_i S^i + w\left(V^i + R^i\right) + c_{i-1} S^{i-1} - c_i S^i \ (i = 2, 3) \end{cases}$$

The infection dynamics is described as follows:

$$\begin{cases} \dfrac{dI_m^i}{dt} = p_m \lambda_i S^i - \gamma_m I_m^i + c_{i-1} I_m^{i-1} - c_i I_m^i \ (i = 1,2,3) \\ \dfrac{dI_s^i}{dt} = p_s \lambda_i S^i - \gamma_s I_s^i + c_{i-1} I_s^{i-1} - c_i I_s^i \ (i = 1,2,3) \\ \dfrac{dR^i}{dt} = \gamma_m I_m^i + \gamma_s I_s^i - wR^i + c_{i-1} R^{i-1} - c_i R^i \ (i = 1,2,3) \end{cases}$$

We assumed that the oldest people have mild symptoms as described above. We assumed that the number of people is stable for a year, implying that death rates in people over 5 years old are equal with birth rates in people aged less than 4 months. Every September, the model adjusts population proportion with the demographic data.

$$\begin{cases} \dfrac{dV^4}{dt} = -wV^4 + c_3 V^3 - B_{\text{year}} \dfrac{V^4}{N^4} \\ \dfrac{dS^4}{dt} = -\lambda_4 S^4 + w(V^4 + R^4) + c_3 S^3 - B_{\text{year}} \dfrac{S^4}{N^4} \\ \dfrac{dI_m^4}{dt} = \lambda_4 S^4 - \gamma_m I_m^4 + c_3 I_m^3 - B_{\text{year}} \dfrac{I_m^4}{N^4} \\ \dfrac{dR^4}{dt} = \gamma_m I_m^4 + c_3 R^3 - B_{\text{year}} \dfrac{R^4}{N^4} \end{cases}$$
